# Predisposing Risk Factors for Delirium in Living Donor Liver Transplantation Patients in Intensive Care Units

**DOI:** 10.1371/journal.pone.0096676

**Published:** 2014-05-08

**Authors:** Szu-Han Wang, Jiun-Yi Wang, Ping-Yi Lin, Kuo-Hua Lin, Chih-Jan Ko, Chia-En Hsieh, Hui-Chuan Lin, Yao-Li Chen

**Affiliations:** 1 Organ Transplant Center, Changhua Christian Hospital, Changhua, Taiwan; 2 Department of Health Care Administration, Asia University, Taichung, Taiwan; 3 Transplant Medicine & Surgery Research Centre, Changhua Christian Hospital, Changhua, Taiwan; 4 Department of Senior Citizen Welfare and Business, Hung Kuang University, Taichung, Taiwan; 5 Department of General Surgery, Changhua Christian Hospital, Changhua, Taiwan; 6 School of Medicine, Chung Shan Medical University, Taichung, Taiwan; 7 School of Medicine, Kaohsiung Medical University, Kaohsiung, Taiwan; D'or Institute of Research and Education, Brazil

## Abstract

**Background:**

Delirium is one of the main causes of increased length of intensive care unit (ICU) stay among patients who have undergone living donor liver transplantation (LDLT). We aimed to evaluate risk factors for delirium after LDLT as well as to investigate whether delirium impacts the length of ICU and hospital stay.

**Methods:**

Seventy-eight patients who underwent LDLT during the period January 2010 to December 2012 at a single medical center were enrolled. The Confusion Assessment Method for the Intensive Care Unit (CAM-ICU) scale was used to diagnose delirium. Preoperative, postoperative, and hematologic factors were included as potential risk factors for developing delirium.

**Results:**

During the study period, delirium was diagnosed in 37 (47.4%) patients after LDLT. The mean onset of symptoms occurred 7.0±5.5 days after surgery and the mean duration of symptoms was 5.0±2.6 days. The length of stay in the ICU for patients with delirium (39.8±28.1 days) was significantly longer than that for patients without delirium (29.3±19.0 days) (p<0.05). Risk factors associated with delirium included history of alcohol abuse [odds ratio (OR) = 6.40, 95% confidence interval (CI): 1.85–22.06], preoperative hepatic encephalopathy (OR = 4.45, 95% CI: 1.36–14.51), APACHE II score ≥16 (OR = 1.73, 95% CI: 1.71–2.56), and duration of endotracheal intubation ≥5 days (OR = 1.81, 95% CI: 1.52–2.23).

**Conclusions:**

History of alcohol abuse, preoperative hepatic encephalopathy, APACHE II scores ≥16 and endotracheal intubation ≥5 days were predictive of developing delirium in the ICU following liver transplantation surgery and were associated with increased length of ICU and hospital stay.

## Introduction

Delirium is a psycho-organic disorder frequently encountered in patients in the intensive care unit (ICU) [1]. Postoperative delirium often occurs in patients after major surgery and has been shown to result in longer hospital stay and a higher rate of complications in the ICU [1], [2]. Clinical features of delirium include impaired consciousness, reduced attention span, disorientation, and rage [1]–[3]. Delirium in patients in the ICU may result in the self-removal of catheters or intravenous tubing or attempts to get out of bed, resulting in falls or injury, which can increase the duration of ICU stay [4]–[6].

Studies have shown that patients with chronic liver diseases or impaired liver function are at increased risk for developing delirium after a surgical procedure [7]. The reported incidence of postoperative delirium is 17% for patients who have undergone liver resection for hepatocellular carcinoma [8] and 10.3% to 26.7% for patients who have undergone orthotopic liver transplantation (OLT) [9], [10].

However, the risk factors for developing delirium in the ICU after living donor liver transplantation (LDLT) have not been well investigated. In this retrospective study, we determined the factors that are predictive of delirium in patients in the ICU after LDLT surgery and investigated whether delirium influences the length of ICU and hospital stay.

## Methods

### Patients

**Figure 1:**
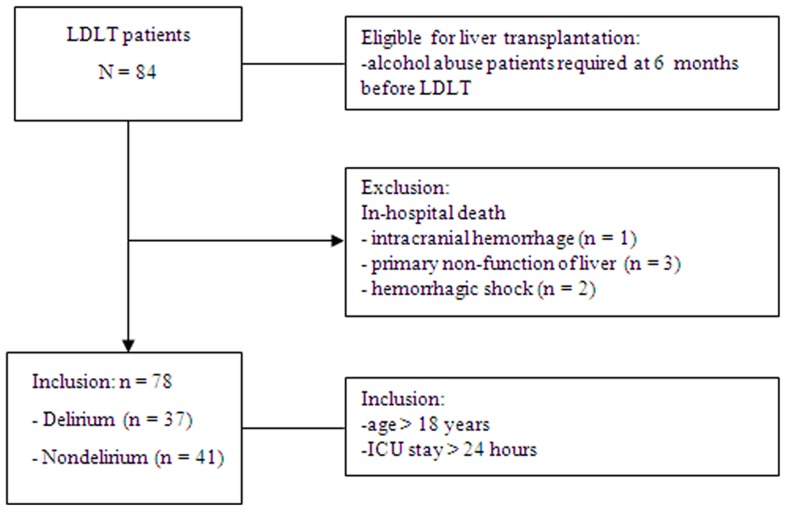
Screening and enrollment.

In this retrospective cross-sectional study, we enrolled patients who had undergone LDLT during the period January 2010 to December 2012 at a single medical center in Taiwan. The inclusion criteria included age >18 years and patients who remained in the ICU for more than 24 hours. Exclusion criteria included in-hospital death due to intracranial hemorrhage, primary non-function of liver or hemorrhagic shock after transplantation (Figure 1). All patients with a history of alcohol abuse abstained from alcohol consumption for at least 6 months before liver transplantation. The medical records of patients in this study were de-identified, analyzed anonymously, and reviewed after patients had signed a consent form. The study was approved by the institutional review board of the Changhua Christian Hospital (Document no. 120706).

### Clinical data

Patient characteristics including gender, age, marital status, disease history (diabetes mellitus or cardiovascular disease), indications for LDLT, history of hypnotic drug use, and history of alcohol abuse were recorded. Preoperative factors included severity of chronic liver disease (assessed using the Model for End-Stage Liver Disease (MELD) scoring system) and diagnosis of preoperative hepatic encephalopathy (assessed and graded using the West Haven Criteria) [11]. Postoperative factors included duration of surgery, volume of blood loss, Acute Physiology and Chronic Health Evaluation II (APACHE II) score, duration of endotracheal intubation, infection status (blood and respiratory tract infection), serum sodium, total bilirubin (TBIL) value, and FK506 (anti-rejection drugs) concentration during ICU stay.

### Assessments

Establishment of a diagnosis of delirium in patients in the ICU requires the use of a sedation assessment tool prior to assessing consciousness, such as the Richmond Agitation-Sedation Scale (RASS) [12]. Patients with a RASS score greater than −4 (−3 through +4) are then evaluated using the Confusion Assessment Method for the Intensive Care Unit (CAM-ICU), a widely-used assessment tool to evaluate the cognitive status of nonverbal and mechanically ventilated patients in the ICU [4]. The scoring system involves systematically moving through four features, namely: 1) acute onset of mental status changes or a fluctuating course, 2) inattention, 3) disorganized thinking, and 4) level of consciousness. Delirium was diagnosed when a patient tested positive for features 1) and 2) and tested positive for either feature 3) or feature 4) [1]. All patients were transferred to the ICU for critical care (1 person per room) after having undergone LDLT. Each patient was cared for by three nurses each day and CAM-ICU evaluation was carried out every 12 hours.

### Immunosuppressive

All patients received calcineurin-inhibitor (CNI)-based immunosuppressive agents. The majority of patients were maintained on tacrolimus in combination with mycophenolate and methylprednisolone. The target level of the CNI-based immunosuppressors administered after the first post-transplant year was 5–10 ng/mL for tacrolimus. The doses of methylprednisolone, initially administered intravenously in 4 divided doses on a daily basis, were tapered from 200 to 20 mg/d over a period of 6 days.

### Statistical analysis

Continuous variables are presented as mean ± standard deviation (SD) and categorical variables are presented as percentages. The Mann-Whitney U-test was used to compare differences in continuous variables including age, MELD score, duration of surgery, volume of blood loss, serum sodium, TBIL values, and FK506 concentrations between patients with delirium and those without the syndrome. The chi-square test was used to compare differences in categorical variables such as gender, marital status, indications for LDLT, history of hypnotic drug use, history of alcohol abuse, evidence of preoperative hepatic encephalopathy, APACHE II score, and duration of endotracheal intubation between the two groups. Significant variables in the univariate analyses were then included in a forward, stepwise multiple logistic-regression model to identify the most important risk factors for developing delirium in the ICU after LDLT surgery. A P-value <0.05 was considered to represent statistical significance. All statistical analyses were performed on a personal computer with the statistical package SPSS for Windows (Version 18, SPSS, Chicago, Il, USA).

## Results

A total of 78 patients (mean age, 53.4±8.4 years) met the inclusion criteria. Of them, 74.4% were male and 82.1% were married. In addition, 42.3% required LDLT for alcoholic liver disease, 57.7% had a history of hypnotic drug use, and 42.3% had a history of alcohol abuse (Table 1).

**Table 1:** Characteristics of patients who underwent living donor liver transplantation.

Characteristics^a^	Delirium	Nondelirium	Total	p-value
	(n = 37)	(n = 41)	(N = 78)	
Age^b^	55.0±7.5	52.0±9.4	53.4±8.4	0.131
Male	27 (73.0)	31 (75.6)	58 (74.4)	0.802
Married	28 (75.7)	36 (87.8)	64 (82.1)	0.238
**Disease history**				
Diabetes mellitus	4 (10.8)	4 (9.8)	8 (10.3)	1.000
Cardiovascular Disease	6(16.2)	2 (4.9)	8 (10.3)	0.141
**Indications for LDLT**				0.022
Alcoholic liver disease	21 (56.8)	12 (29.3)	33 (42.3)	
Viral hepatitis	14 (37.8)	28 (68.3)	42 (53.8)	
Primary biliary cirrhosis	2 (5.4)	1 (2.4)	3 (3.9)	
Hypnotic drug use	25 (67.6)	20 (48.8)	45 (57.7)	0.112
Alcohol abuse history	21 (56.8)	12(29.3)	33 (42.3)	0.021

a Data are shown as n (%) and were compared using the chi-square test.

b Data are shown as mean ± standard deviation and were compared using the Mann–Whitney test.

Delirium was diagnosed in 37 (47.4%) patients in the ICU after LDLT. Of these patients, approximately 50% developed symptoms of delirium within 5.5 days after surgery, with symptoms lasting for a mean of 5.0±2.6 days. Patients with delirium had a higher rate of alcohol abuse history (56.8% vs. 29.3%) and preoperative hepatic encephalopathy (62.2% vs. 24.4%), higher APACHE II scores (≥16) (71.3% vs. 36.6%), longer duration of endotracheal intubation (≥5 days) (40.5% vs. 14.6%), and higher rates of infection (43.2% vs. 24.4%) (p<0.05) than patients without delirium. In addition, the length of stay was significantly higher in patients with delirium than in patients without the syndrome (39.8 days vs. 29.3 days; p<0.05) (Table 2).

**Table 2:** Clinical parameters and outcomes of patients.

Parameters^a^	Delirium	Nondelirium	Total	p-value
	(n = 37)	(n = 41)	(N = 78)	
Preoperative hepatic encephalopathy^b^	23 (62.2)	10 (24.4)	33 (42.3)	0.001
MELD score	19.8±8.3	15.9±7.9	16.2±7.7	0.071
Blood loss (mL)	5066±4733	4810±3545	4994±4187	0.786
Duration of surgery (min)	483.5±89.8	503.5±108.5	488.8±98.5	0.382
**APACHE II score** b				0.003
<15	11 (29.7)	26 (63.4)	37 (47.4)	
≥16	26 (70.3)	15 (36.6)	41 (52.6)	
**Endotracheal intubation (day)** b				0.012
<4	22 (59.5)	35 (85.4)	57 (73.1)	
≥5	15 (40.5)	6 (14.6)	21 (26.9)	
Infection^b^	16 (43.2)	10 (24.4)	26 (33.3)	0.043
TBIL (mg/dL)	6.7±3.9	5.3±3.0	5.6±2.8	0.071
Serum sodium (mmol/L)	140±7.2	137±9.1	138±8.1	0.545
FK 506 concentration (ng/mL)	6.0±2.9	6.5±2.2	6.4±2.5	0.378
Duration of ICU stay (day)	15.4±19.4	10.2±5.7	11.4±5.4	0.105
Length of stay (day)	39.8±28.1	29.3±19.0	30.4±18.0	0.034

a Data are shown as mean±standard deviation and were compared using the Mann-Whitney test.

b Data are shown as n (%) and were compared using the chi-square test. MELD score, the model of end-stage liver disease score; APACHE II score, Acute Physiology and Chronic Health Evaluation II score; TBIL, Total bilirubin.

Significant preoperative risk factors for delirium in the ICU after LDLT included history of alcohol abuse (Odds Ratio [OR] = 6.40, 95% Confidence Interval [CI]: 1.85–22.06) and preoperative hepatic encephalopathy (OR = 4.45, 95% CI: 1.36–14.51). Significant postoperative risk factors included APACHE II score ≥16 (OR = 1.73, 95% CI: 1.71–2.56) and duration of endotracheal intubation ≥5 days (OR = 1.81, 95% CI: 1.52–2.23) (Table 3).

**Table 3:** Factors associated with the development of delirium in the ICU after living donor liver transplantation.

Parameters	odds ratio	95% CI	p-value
Alcohol abuse history	6.40	1.85–22.06	0.003
Preoperative hepatic encephalopathy	4.45	1.36–14.51	0.013
APACHE ΙΙ score	1.73	1.71–2.56	0.007
Endotracheal intubation (day)	1.81	1.52–2.23	0.037

## Discussion

In this study, 47% of the patients who underwent LDLT developed delirium in the ICU. The incidence of delirium in our patients was higher than that reported in patients in the ICU after OLT (10%∼26.7%) [9], [10]. The incidence of delirium, therefore, appears to increase with the complexity of surgical procedures and severity of disease. To the best of our knowledge, this is the first study to investigate the risk factors for developing delirium in the ICU after LDLT. In our transplant center, the incidence of alcoholic liver disease among patients who underwent LDLT (42.3%) was more than two-fold higher than that among patients who underwent OLT (21.4%) [9]. In addition, the incidence of preoperative hepatic encephalopathy in our study (62.2%) was also higher than that reported in patients who underwent liver transplantation (51.5%) [13]. These factors might have contributed to the high incidence of delirium in our study.

Previous studies have shown that post-surgical delirium can result in increased postoperative morbidity and prolonged ICU and hospital stay [6], [14], [15]. In this study, we found that patients who developed delirium after LDLT spent an average of five more days in the ICU and 10 more days in the hospital than patients who did not show symptoms or signs of delirium.

History of alcohol abuse is a well-known risk factor for postoperative delirium [16]–[18]. In this study, approximately 42% of all patients had a history of alcohol abuse; however, the prevalence of alcoholism was significantly higher among patients who developed delirium in the ICU than among those who did not (57% vs 29%).

Hepatic encephalopathy is a specific neurological symptom in patients with liver function disorders and can be sub-divided into minimal, episodic, and persistent encephalopathy. Clinical manifestations include various states of confusion and behavioral changes, which might predispose patients to increased risk for postsurgical delirium in the ICU [19], [20]. In this study, we found that the risk of developing delirium in the ICU after LDLT was more than 3 times higher in patients who were admitted with hepatic encephalopathy than in other patients.

Several studies have shown that high-volume blood loss during surgical procedures is a risk factor for developing delirium in the ICU [21]–[23]. In this study, however, blood loss was not a significant risk factor for postoperative delirium. It has also been reported that blood transfusion, renal replacement therapy, and APACHE II score are associated with the development of postoperative delirium in patients in the ICU after OLT [9]. In this study, we found that an APACHE II score ≥16 and prolonged endotracheal intubation were predictive of postoperative delirium in the ICU after LDLT. In fact, we found that patients who required prolonged endotracheal intubation (≥5 days) were at almost twice (OR 1.81) the risk of developing delirium than patients who were intubated for less than 5 days. However, the mechanism underlying the development of postoperative delirium is controversial and clinical risk factors for developing delirium in the ICU after LDLT have not been well investigated. To the best of our knowledge, this is the first study to assess the potential risk factors for developing delirium in the ICU after LDLT. The limitations of our study include potential biases inherent to the nature of any retrospective study and its small sample size.

## Conclusions

History of alcohol abuse, evidence of preoperative hepatic encephalopathy, APACHE II score ≥16, and prolonged endotracheal intubation are associated with the development of delirium in the ICU following living donor liver transplantation surgery and are associated with increased length of ICU and hospital stay.
